# Validation and application of the personnel factor for the garment used in cleanrooms

**DOI:** 10.1016/j.dib.2015.12.031

**Published:** 2016-01-02

**Authors:** Shih-Cheng Hu, Angus Shiue

**Affiliations:** National Taipei University of Technology, Taiwan

## Abstract

The cleanroom environment has many potential sources of contamination, including: operators, equipment, structures, and any surface that can create particles via friction, heat, exhaust, outgassing, and static electricity charge. Operatives working in the cleanroom are the major source of particles. While cleanroom operators work, they emit millions of particles from every activity. Particles migrate up the cleanroom garment to the head and drop to the legs during cleanroom movements. Specialized textile fabrics have been used in cleanroom garments for many years. The need for this type of fabric has increased mainly due to the need to protect critical operations in cleanrooms as well as creating comfort for operators and other personnel. This study covers the general static wind-driven method, the Helmke Drum method and the dispersal chamber to measure particle penetration, shedding, and generation, in regards to the filtration efficiency of cleanroom fabrics and garments. Firstly, particle penetration is shown to increase with increasing face velocity and decreasing particle size below 1 μm. Secondly, that a recommended upper particle-size limit should be 5 µm. Using the Helmke drum test, the size distribution of particles released from the garment is shown to follow a power law distribution, with a slope of less than 1. Furthermore, the study introduces dynamic body box for testing fabrics as well as cleanroom garments. It is more practical and sensitive when compared to traditional methods and is based on a more concise technical approach. The life-time cycle performance of a typical cleanroom garment coverall is examined, particularly looking at the implications of pre-use steralization.

**Specifications Table**

**Table:** 

Subject area	Physics
More specific subject area	Pharmaceutical/Biotech Cleanroom
Type of data	Table: 5, figure: 8
How data was acquired	MetOne 237B and MetOne 2100 particle counter
Data format	Raw data
Experimental factors	Brief description of any pretreatment of samples
Experimental features	Particle Penetration Test, Particle Shedding Test-Helmke Drum Tumble Test, Dispersal Chamber Test
Data source location	Air System Enterprise Co., LTD. Taiwan, ROC.
Data accessibility	Correlation coefficients of particle concentration data detection is over 0.9258

**Value of the data**

• The increasing cleanliness demands regarding the performance of modern cleanroom clothing systems.
• Particle penetration increases with increasing face velocity and decreases particle size below 1 μm.
• The size distribution of particles released to follow a power law distribution with a slope of less than 1.
• Body box for testing the efficiency of the cleanroom garments is more practical and sensitive.

## 1 Data

### 1.1 Experimental design, materials and methods

#### 1.1.1 Materials

The garment tested in this study is 98% polyester filament yarn+2% conductive yarn. The characteristics of the garment are listed in Table 1.

#### 1.1.2 Particle penetration test

**Fig. 1:**
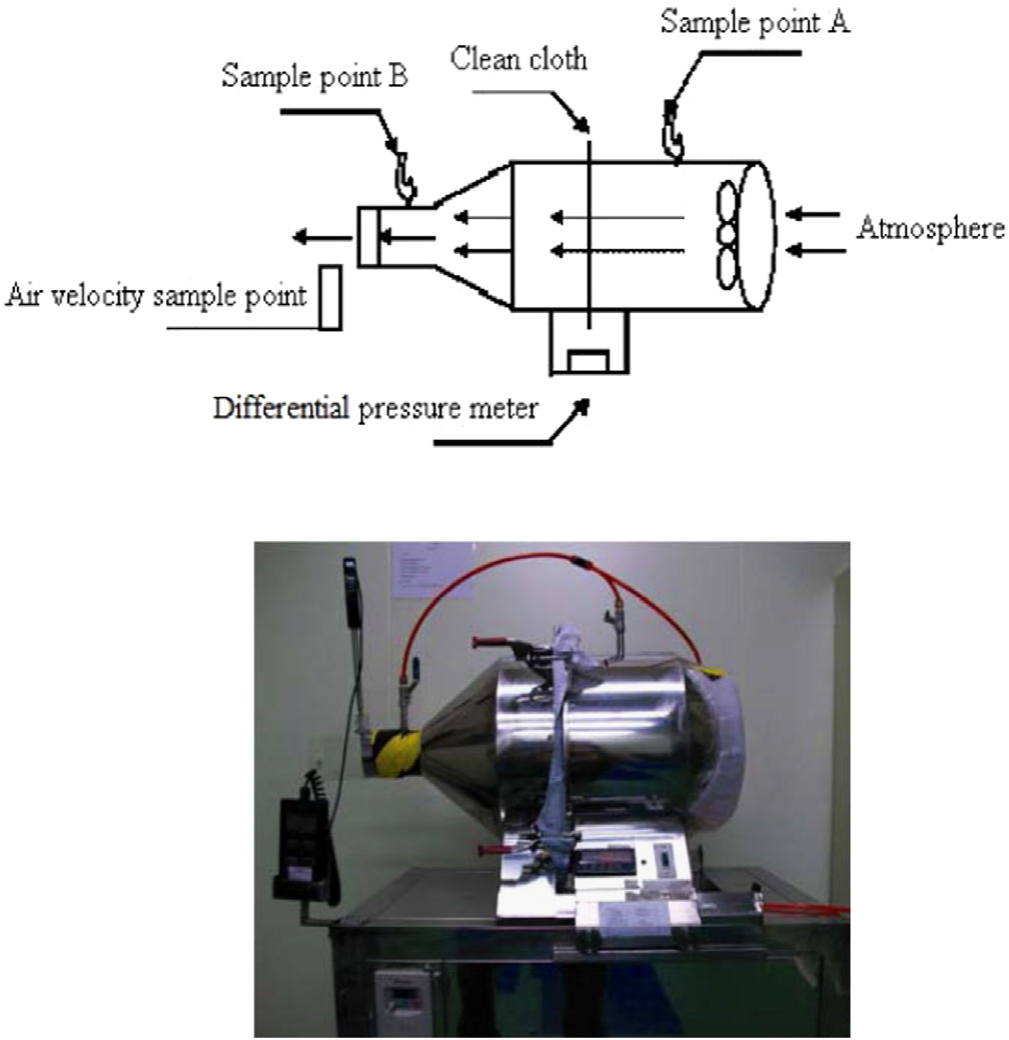
Schematic diagram of particle penetration test apparatus.

The apparatus was set up in a Class-10 modular cleanroom utilizing the ambient aerosol as the encounter. The test fabric was installed in a filter container which had a 25-cm (10-in.) diameter dynamic filtration area. A vacuum pump was utilized to set flow via the fabric at a rate that yielded a pressure drop of 9.5 mm H_2_O. The aerosol particle counter was utilized to successively get ten 1-min upstream and ten 1-min downstream samples. From the particle counter data (MetOne 237B: ±10% accuracy, 0.3 µm (237B) at 0.1 CFM (2.83 LPM) flow rate, linked to a manifold with multichannel; Table 2), the filtration efficiency of the media was calculated for two size ranges: 0.1 μm and >5 μm. The test was replicated, and a second set of filtration efficiency values were calcula ted. If the efficacy values from the two sets were not in 15%, the test would be replicated until two values reach the goal to be within 15%. The average of the two efficacy values was then calculated and noted. The arrangement is shown schematically in Fig. 1.

The particles flow through the garment, if the upstream concentration of particles is *C_u_* and the downstream concentration of particles is *C_d_*, the penetration of particles is determined as:

(1) $$P=\frac{C_{d}}{C_{u}}$$

#### 1.1.3 Particle Shedding Test–Helmke Drum Tumble Test

**Fig. 2:**
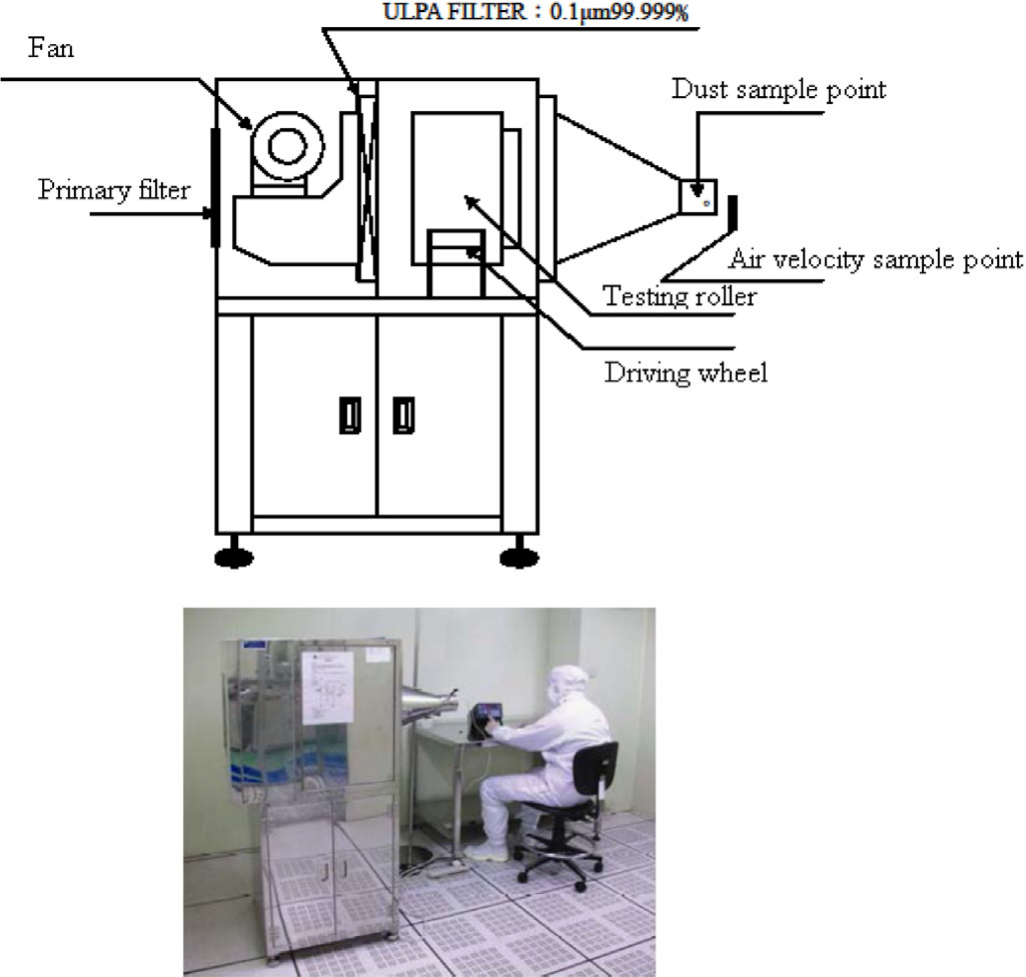
Schematic diagram of Helmke Drum test method.

The apparatus was set up in a Class-10 modular cleanroom. The end of the sampling tube for an airborne particle monitor was mounted to pull the air from the inside of the rotating drum. The number of airborne particles was determined utilizing a particle counter (MetOne 237B: Table 2). The arrangement is shown schematically in Fig. 2.

A power law distribution is given by the following equation [13]:

(2) $$N(d)=Ad^{\left(-B\right)}$$

where: *N*(*d*) is the cumulative concentration as a function of particle diameter, *d* is the particle diameter, and *A* and *B* are statistically-determined coefficients.

#### 1.1.4 Dispersal Chamber Test

**Fig. 3:**
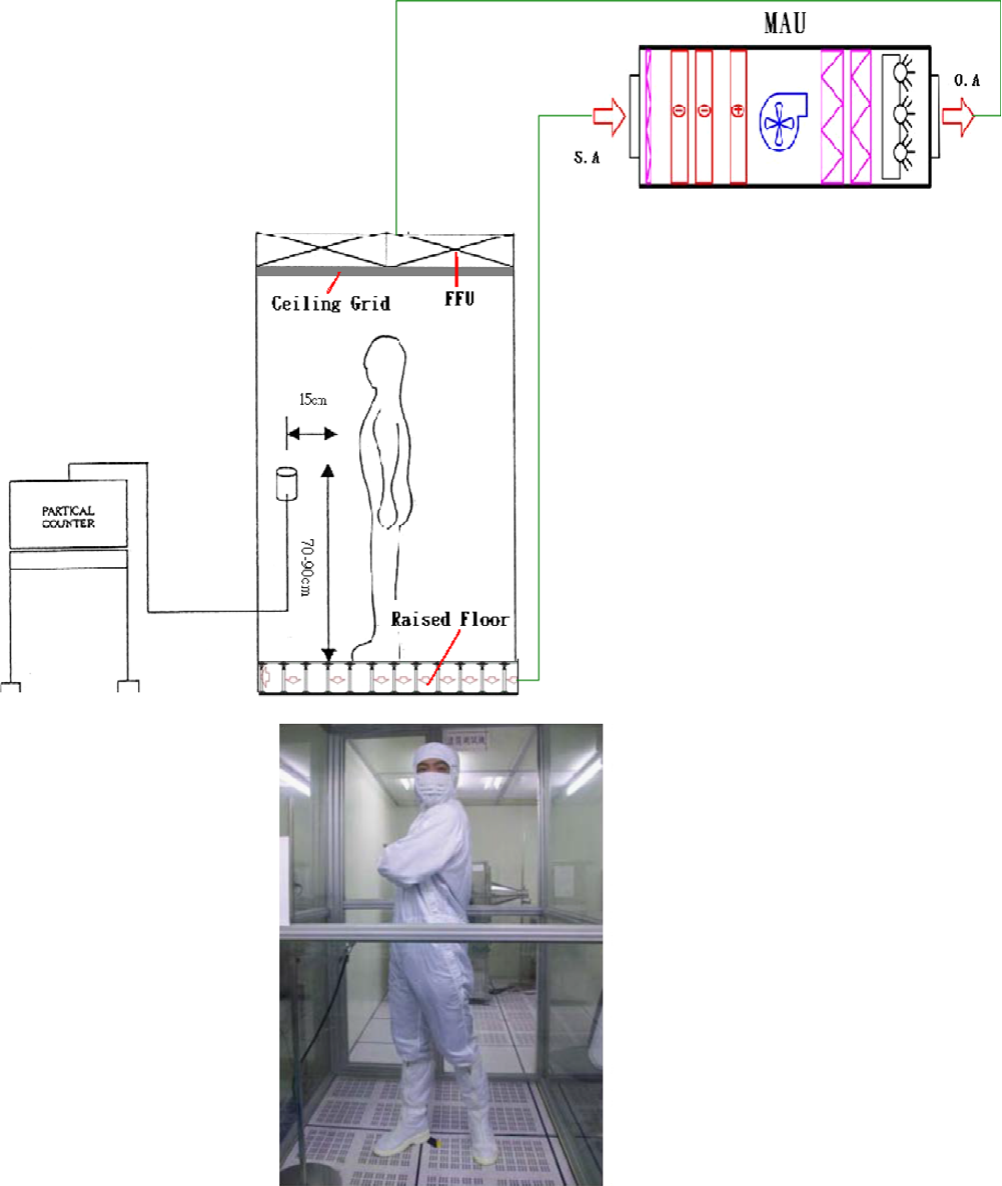
Principal arrangement of dispersal chamber (body-box).

A specially designed dispersal chamber (120 cm(L)×120 cm(W)×310 cm(H)) with HEPA-filtered air supply and separate make-up air unit has been deemed suitable for the evaluation of clean room clothing systems. The apparatus was set up in a Class-1 modular cleanroom. The arrangement is shown schematically in Fig. 3. The vertical unidirectional air velocity was adjusted at 0.35 m/s and the dispersal chamber is pressurized relative to the adjacent area. Room temperature and relative humidity are not controlled since the indoor environment was reasonably stable, with 23±3 °C and 25–55% RH during the tests. The total number of airborne particles was determined using a particle counter (MetOne 2100: ±10% accuracy, 1.7 m^3^/h sampling airflow rate, linked to a manifold with multichannel; Table 3) and particles were gathered primarily utilizing a slit-sampler (brand name FH3). In some cases, they were additionally measured utilizing a sieve-sampler (Andersen 6-stage Sampler).

#### 1.1.5 The test person: female, 58 kg, 160 cm tall, with long black hair

Cleanroom garment: A coverall and hood (100% polyester), single use facial protection and latex gloves. The coverall and hood were produced in a cleanroom environment which were new and had been subjected to washing prior to use.

#### 1.1.6 Movement

*Standing while performing arm movements*: One arm at a time was moved at an angle of 90°, back and forth in a sweeping motion. The original position of the arm was directed straightly ahead with a 90° bend at the elbow. The movement frequency was one second for one arm to move back and forth.

*Standing with cross beat*: Both hands beat the chest from side to side, as far as possible in each direction. The time for turning from one side to the other was one second.

*Standing with rotating torso*: Both hands grabbing the waist and rotating the upper body from side to side, as far as possible in each direction. The time for turning from one side to the other was one second.

#### 1.1.7 Walking on the spot: walking on the spot at a rate of two steps per second

If the contamination sources and the design of the dispersal chamber system are known, a mathematical model can be constructed of the level of airborne contaminants in a dispersal chamber having fully turbulent mixing air. The assumptions is no leakage into the dispersal chamber and an approach to 100% efficiency from HEPA filters, the simplest possible expression defining the concentration *c* in the dispersal chamber is as following given by Ljungqvist B and Reinmüller [26], [27], [28]:

(3) $$c=\frac{q_{s}}{Q}$$

where *q_s_* is source strength of outward particle flow (numbers/s), and *Q* is the total airflow (m^3^/s).

## 2 [(NO) Conclusions/Summary

Validation and application of the personnel factor for the anti-static electric garment used in cleanrooms.

## Figures and Tables

**Table 1:** The characteristics of the garment.

Composition	98% Polyester filament yarn+2% conductive yarn		
Weave	2/2 twill, mm Grid		
Density	Warp	188 ends/in. (75 ends/cm)±5%	
Weft	114 ends/in. (44 ends/cm)±5%
Weight	g/m**^2^**	109±5%	
Yarn type	Warp	Polyester 75D/72F+	
Conductive Yarn
Weft	Polyester 75D/72F+
Conductive Yarn
**Air Permeability**	**cc/cm^2^/s**	**4.93**	**JIS L1096-A-1990**
**Surface Resistivity**		**10**^**5–6**^**(42%R.H., 21 °C) ohm/square**	**DIN 54345**
Friction Charges	Warp	43 V	JIS L1094-B
Weft	22 V
Decay Time		±0.01(42%R.H., 21 °C)	NFPA-99
Half Life	Warp	1 s	JIS L1094-4
Weft	1 s
Tensile Strength	Warp	100.0 kg	JIS L1096.6.12.1-A
Weft	66.3 kg
Tear Strength	Warp	2807 g	JIS L1096.6.15.5-D
Weft	1917 g
Wash Shrinkage	Warp	−1.20%	AATCC 135-1V-1995

**Table 2:** Specification of MetOne 237B particle counter.

Title	Values
Size Channels	0.3, 0.5, 7.0, 1.0, 2.0, 5.0 μm
Flow Rate	0.1 cfm
Sample Time	1 s to 24 h
Hold Time	1 s to 24 h
Location Labels	250 appear on printout
Datalogging	500 samples, rotating buffer
Output	Built-in printer, RS-232 port
Power	Rechargeable Ni–Cd battery, 4 h operation with printer, 8 h without or AC operation with adapter/charger

**Table 3:** Specification of MetOne 2100 particle counter.

MODEL	2100
Smallest Size, μm	0.1
Number of Channels	6
Flow Rate, cfm	1
Flow Rate, L/min	28.3
Laser Type	4-port HeNe
Communication Support	RS-232
RS-485	
Optional	RH/Temp
Environmental Probes	Air Velocity special:dP
Coincidence Loss 5%	40,000
(counts/ft3)	
Display Type/Digits	Red LED
Memory Buffer Records	400
Location Labels	1000
Printer Support	Built-in or External
Vacuum Source	AC; Oscillating
Size, *W*×*H*×*D*, in.	13.5×7.0×22
Size, *W*×*H*×*D*, cm	34×18×57
Weight, lbs/kg	42 lbs (19 kg)
Accessories Included	Isokinetic Probe w/ Tripod Zero Count Filter
